# Surface-sampling mass spectrometry to study proteins and protein complexes

**DOI:** 10.1042/EBC20220191

**Published:** 2023-03-29

**Authors:** Kei F. Carver Wong, Rebecca E. Greatorex, Charlotte E. Gidman, Sidrah Rahman, Rian L. Griffiths

**Affiliations:** School of Pharmacy, University of Nottingham, Nottingham NG7 2RD, U.K.

**Keywords:** imaging techniques, mass spectrometry, proteomics, surface analysis

## Abstract

This review aims to summarise the current capabilities of surface mass spectrometry (MS) approaches that offer intact protein analysis, and that of non-covalent complexes. Protein analysis is largely achieved via matrix-assisted laser desorption/ionisation (MALDI), which is in itself a surface analysis approach or solvent-based electrospray ionisation (ESI). Several surface sampling approaches have been developed based on ESI, and those that have been used for intact protein analysis will be discussed below. The extent of protein coverage, top-down elucidation, and probing of protein structure for native proteins and non-covalent complexes will be discussed for each approach. Strategies for improving protein analysis, ranging from sample preparation, and sampling methods to instrument modifications and the inclusion of ion mobility separation in the workflow will also be discussed. The relative benefits and drawbacks of each approach will be summarised, providing an overview of current capabilities.

## Proteins and protein complexes

Proteins play a fundamental role in biological processes and can regulate normal functions that are altered in disease. Top-down analysis of intact proteins and native MS offer opportunities to maintain and therefore probe not only primary, secondary, and tertiary structure but can also allow study of quaternary structure for the case protein–ligand complexes. Furthermore, modifications such as amino acid substitutions can be lost during enzymatic digest analysis, as only parts of the protein are analysed. The desire to probe protein structure is combined with a growing interest in understanding their spatial distributions within tissues, which is afforded by many surface MS approaches. This review seeks to evaluate current surface analysis mass spectrometry and imaging of intact proteins and non-covalent complexes. The analysis of digested proteins has not been included, the following review may be of interest for *in situ* analysis of peptides and/or digested proteins alongside intact proteins [1]. Fragmentation experiments via tandem mass spectrometry (MS/MS) will be commented on herein. Protein fragmentation provides primary amino acid sequence information, with percentages showing the extent of peptide bonds broken. For non-covalent complex, MS/MS can indicate components by dissociating weak interactions between proteins and/or ligands. Freshly frozen or untreated tissue samples are typical for protein analysis as formalin fixation cross-links proteins and therefore intact proteins cannot be analysed. Tissue washing protocols can be used to remove, for example, abundant lipids.

## Matrix-assisted laser desorption/ionisation (MALDI)

**Figure 1:**
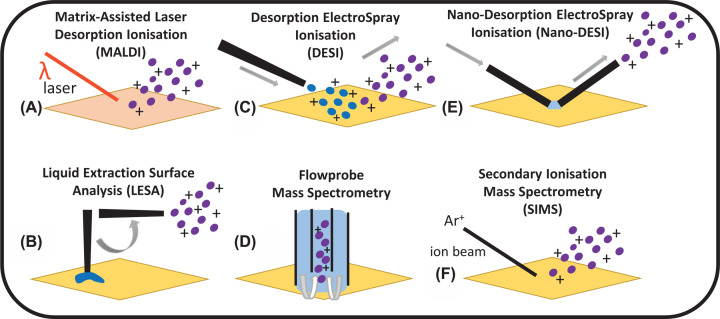
Schematics of some of the commonly utilised surface-sampling mass spectrometry and imaging techniques for protein analysis (**A**) MALDI, (**B**) LESA, (**C**) DESI, (**D**) Flowprobe MS, (**E**) Nano-DESI, (**F**) SIMS.

**Figure 2:**
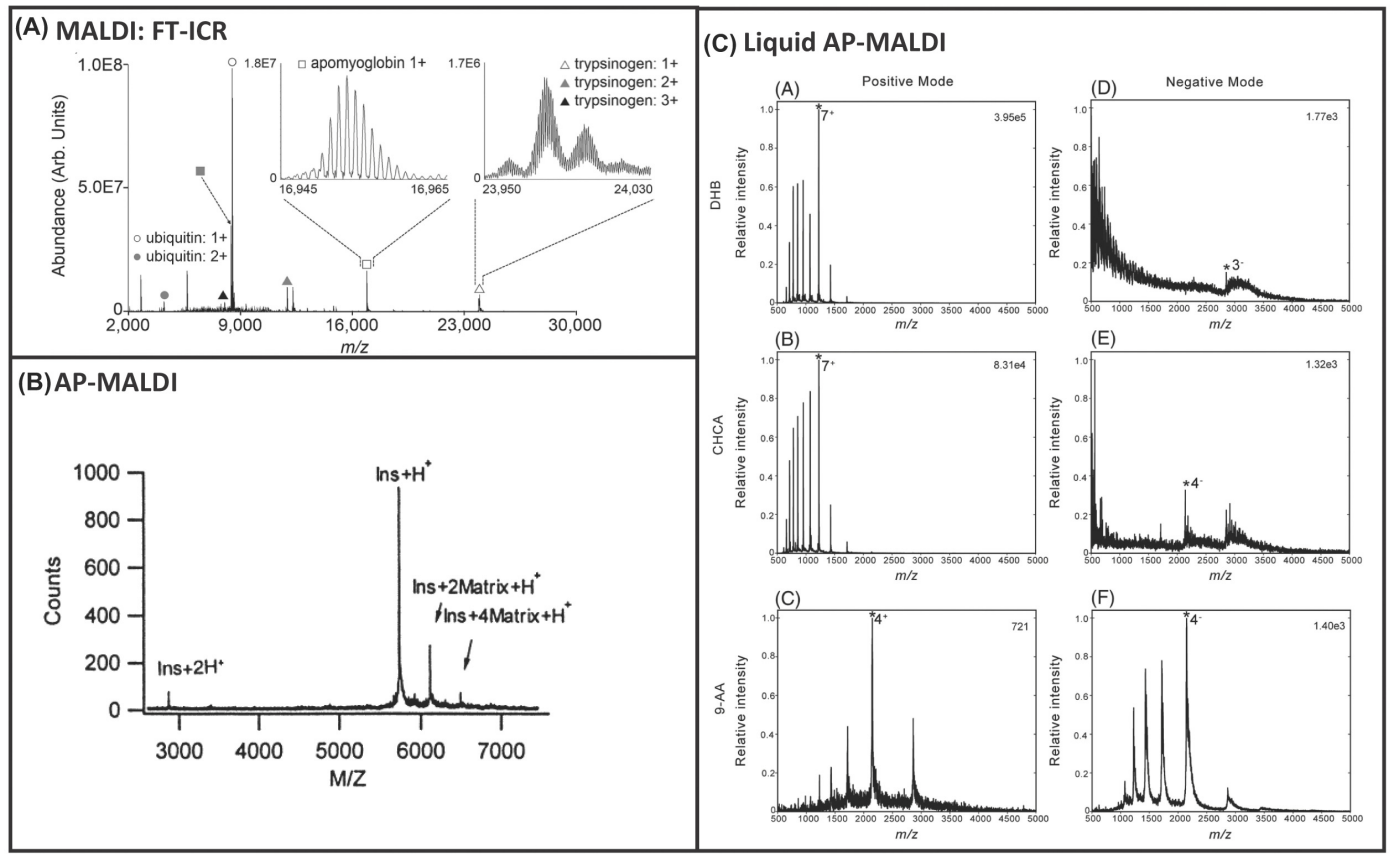
Matrix-Assisted Laser Desortion/Ionisation (MALDI) of peptides and proteins (**A**) MALDI FTICR of peptides reprinted with permission from Prentice et al., Enhanced Ion Transmission Efficiency up to *m*/*z* 24 000 for MALDI Protein Imaging Mass Spectrometry, Analytical Chemistry, Copyright 2018, American Chemical Society, (**B**) AP-MALDI of intact proteins reprinted with permission from Laiko et al., Atmospheric Pressure Matrix-Assisted Laser Desorption/Ionisation Mass Spectrometry, Analytical Chemistry, Copyright 2000, American Chemical Society, and (**C**) Liquid AP-MALDI of intact proteins reprinted with permission from Hale et al., Production and analysis of multiply charged negative ions by liquid atmospheric pressure matrix-assisted laser desorption/ionisation mass spectrometry, Rapid Communications in Mass Spectrometry, 2018, open access through a Creative Commons Licence.

This approach uses a matrix compound that absorbs (UV) laser energy that is irradiated at a surface and transfers this to biomolecules of interest, see Figure 1A. Since the introduction of MALDI in the late eighties, demonstrating intact protein analysis up to 67 kDa using UV lasers [2], its use has become commonplace for surface analysis and imaging of biomolecules. Although the detection of intact proteins up to 150 kDa was reported by Karas and Hillenkamp [3], the analysis of proteins and non-covalent complexes was limited to time-of-flight analysers that have lower resolution than trapping-based counterparts. Thus, electrospray was the ionisation method of choice as multiply charged ions are easily formed and can be analysed via high accurate mass instrumentation and provides better MS/MS data, with superior sequence coverage. MALDI benefits from being a soft ionisation method with high analytical sensitivity, low sample consumption, and high tolerance to contaminants, such as trifluoroacetic acid (TFA). Mass spectrometry imaging (MSI) was first reported by Caprioli et al. for proteins up to 25 kDa [4], and imaging of proteins up to 12 kDa has since been reported at a spatial resolution of 50 µm resolution on a fourier-transform ion cyclotron resonance (FT-ICR) analyser [5]. Poly-peptides have also been studied on similar instrumentation [6]. Instrument adaptations to improve protein transmission have been reported, for imaging of proteins up to 24 kDa on an FT-ICR instrument [7], see Figure 2A. Yet, MALDI of intact proteins remains challenging owing to limitations arising from mass analyser selection and the fact that weak non-covalent interactions can be broken upon laser irradiation and nonspecific clustering. Nevertheless, protein standards up to 330 kDa have been analysed via (traditional) UV-based MALDI lasers using sinapinic acid (SPA) matrix [8,9] and non-covalent protein complexes up to 60 kDa via MALDI using IR-based lasers [10], and up to 50 kDa using liquid matrices [11] (further details below, mixtures of glycerol with common MALDI matrices).

### Atmospheric pressure matrix-assisted laser desorption/ionisation (AP-MALDI)

In 2000, atmospheric pressure MALDI (AP-MALDI) was developed, which has many features in common with conventional vacuum MALDI [12]. Both techniques produce gas-phase ions via a process of laser beam desorption/ionisation of a solid-state target material [12]. Though the sensitivity of AP MALDI is lower than its vacuum counterpart, there is also a reduction in background noise [13]. AP-MALDI has seen widespread use for small molecules (lipids and peptides). Whilst intact proteins up to 6 kDa were analysed by Laiko et al. [12] as shown in Figure 2B, little progress has been made with conventional matrices for intact proteins, rather than digested samples. There are two main commercial AP-MALDI products available: the Mass Tech™ ion source [14] and the Spengler source [15]. The key difference between the two is that the Spengler source also allows 3D depth profiling information to be acquired, similarly to SIMS (see below). The Mass Tech™ ion source can achieve 5 µm, and the Spengler source 1.4 µm spatial resolution [16], enabling detection of small peptides in the outer rim of the cell surface [16]. These sources are not integrated, and are compatible with coupling to numerous vendor instruments, offering greater choice of mass analysers and therefore coupling to, for example, trapping technology. The capability to couple AP-MALDI sources with tandem mass spectrometers facilitates the effective sequencing of proteins [13], benefitting from adjustment of the target plate potential, laminar flow chamber diameter and length, temperature and gas flow [13]. Using an QqLIT (Quadrupole Linear Ion Trap) instrument, MS/MS can be performed on peptides at concentrations as low as 1 fmol [13]. With respect to intact protein and native protein analysis, most progress has been made using a variant to the approach known as liquid AP-MALDI.

### Liquid atmospheric pressure matrix-assisted laser desorption/ionisation (Liquid AP-MALDI)

Vacuum-based and AP-MALDI typically generate singly charged ions. Liquid supports (glycerol and similar compounds that remain liquid) were first introduced for intact protein analysis up to 66 kDa via traditional MALDI (singly charged ions on TOF instrumentation) [17]. Recently, multiply charged ESI-like ions have been generated by AP-MALDI when using a combination of traditional and liquid (e.g., glycerol) matrices [18,19]. The mechanism of ionisation has been hypothesised to be similar to that of laserspray (refer to [20] for more details) or ESI [21]. Early reports of multiply charged ion production were first achieved using glycerol-based liquid samples in IR-MALDI to detect peptides and proteins [22]. Cramer et al. expanded upon these studies showing the benefits of using liquid matrices in IR- and UV-based AP-MALDI MS, namely the stable analyte ion yield and capability to hold additives in the matrix to alter the sample properties [23]. Additional studies have similarly reported the generation of multiply charged ions using liquid AP-MALDI matrices that crucially contain a non-volatile viscous support liquid, like glycerol [24–26] promoting generation of multiply charged analyte ions, enabling detection of peptides, intact proteins up to 80 kDa and protein complexes up to 63 kDa. Multiply charged ions are additionally promoted by ion source parameters such as a heated transfer capillary, gas flow conditions and laser settings, specifically changes in the ionisation interface/ion transfer region. Koch et al. detected protonated species of proteins up to 17 kDa using a glycerol/TFA matrix and a modified AP-MALDI ion source coupled to a QTOF-MS [13]. They noted that the abundance of Na^+^ or K^+^ adducts were low, and that matrix-derived ion signals were detected in the low m/z ranges at low intensity. Ait-Belkacem et al. characterised in-source decay (ISD) and pseudo-MS3 fragment ions of proteins up to 12 kDa using glycerol-based liquid MALDI matrices and an AP-MALDI source coupled to a Q-Exactive Plus [25]. 2,5-DHB liquid matrices provided the highest sequence coverage; 68% for thymosin β4 (5 kDa), and 40% for cytochrome *c* (12 kDa).

Cramer et al. have studied a range of diols and triols as viscous support liquids and traditional and non-traditional matrix chromophores [15], replacing glycerol with ethylene glycol as the liquid support yielded a well-resolved spectrum of BSA (64 kDa). Greater analyte ion signals and signal-to-noise ratios (*S*/*N*) could be achieved using smaller molecules as a liquid support, such as ethylene glycol and propylene glycol, facilitating detection of proteins up to 80 kDa. α-Cyano-hydroxy-cinnamic acid (CHCA) remained an optimal chromophore in positive ion mode, whilst 9-aminoacridine (9-AA) was recommended for negative ion mode studies [27], see Figure 2C. Liquid matrices have only recently been explored for native AP-MALDI MS, which aims to retain intact non-covalent interactions of macromolecules during MS. The performance of liquid AP-MALDI for protein analysis is heavily dependent on efficient optimisation of the MALDI sample conditions and MS instrumental parameters; a binary matrix solution comprising two compounds, one acidic and one basic matrix compound in glycerol that is vacuum stable is recommended. Laser irradiation patterns were also critical [11]. As such, a standardised liquid AP-MALDI workflow for proteomics and successful MS-imaging has yet to be achieved. With regards to protein surface analysis, liquid AP-MALDI can provide a strong alternative to ESI-based approaches with the additional benefits of high sensitivity, low sample consumption, and high tolerance to contaminants.

## Electrospray ionisation (ESI)-based approaches

During ESI analyte ions are sprayed from a solution into the instrument inlet, with a voltage applied at the point of spraying. ESI forms multiply charged ions which is particularly advantageous for the analysis of larger biomolecules such as proteins and non-covalent complexes, thus it is widely used in the biochemical field. It facilitates analysis on high resolution, Orbitrap based, instrumentation and offers improved sequence coverage via MS/MS as more fragment ions are charged rather than neutral. However, as a liquid-based approach the move towards surface analysis has been slow and divergent. Several surface-sampling approaches have been successfully utilised for intact protein analysis and that of non-covalent complexes. The concept of ion mobility separation is introduced in this section, please refer to the following review for further details [28,29].

### Liquid extraction surface analysis (LESA)

Surface sampling is achieved using a robotically operated conductive pipette tip that allows discrete sampling via a liquid junction [30], see Figure 1B. Solvent systems can be tailored to allow extraction of different analytes, or for proteins, under denaturing or native conditions. This approach has been the most studied approach to date for intact proteins and non-covalent complexes, most likely owing to its sensitivity.

Direct analysis of intact proteins was first reported in 2011 for the detection of common haemoglobin variants from dried blood spot cards [31], with unknown variants and thalassemia determined via top-down MS/MS [32,33]. Analysis of intact proteins directly from bacterial colonies grown on media has also been described from microbes including *Escherichia coli* [34], *Staphylococcus aureus* [35], and ESKAPE pathogens cultured in a wound infection model [36,37]. Contact-LESA, where the pipette tip contacts the bacterial colony, was shown to be particularly beneficial for protein extraction [34], notably a high ratio of formic acid was particularly beneficial for gram-positive bacterium [35]. In each of these studies top-down MS/MS allowed identification of 24 proteins; five from *Enterococcus faecium*, four from *Enterococcus* *faecalis*, six from *Klebsiella pneumoniae*, four from *Acinetobacter baumannii*, and five from *Enterobacter cloacae*. The inclusion of ion mobility separation, such as (planar geometry) high-field asymmetric waveform ion mobility (FAIMS), enables molecular separation that filters out lipid and background media signals to simultaneously improve sensitivity for proteins [38]. Direct analysis of yeast proteins has proven challenging via conventional LESA methods, however electroporation prior to LESA MS to lyse cells is recommended; this enabled top-down identification of 10 different proteins each from *Saccharomyces cerevisiae*, *Candida glabrata*, and *Cryptococcus neoformans* [39], many of which had not been reported in their intact form previously. One of the challenges for all surface analysis approaches is the lack of quantitative data, and how to address this challenge. Tissue mimetics have been described for intact protein quantitation from brain tissue [40]; however, one mimetic is typically required for each protein which is expensive and time-consuming. This challenge of quantitation remains within the field.

**Figure 3:**
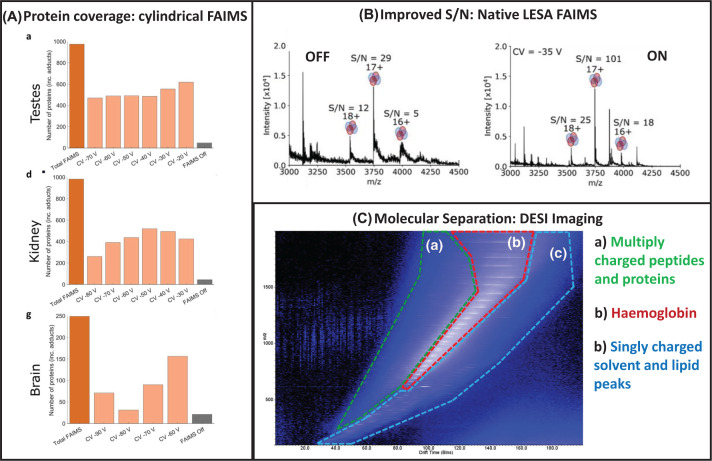
The benefits of ion mobility separation (**A**) Improved protein coverage via LESA FAIMS MS, reprinted with permission from Griffiths et al., Comprehensive LESA Mass Spectrometry Imaging of Intact Proteins by Integration of Cylindrical FAIMS, Analytical Chemistry, Copyright 2020, American Chemical Society. (**B**) Improved signal-to-noise ratios of protein complexes via LESA FAIMS MS, reprinted with permission from Hale et al, High-Field Asymmetric Waveform Ion Mobility Spectrometry and Native Mass Spectrometry: Analysis of Intact Protein Assemblies and Protein Complexes, Analytical Chemistry, Copyright 2022, American Chemical Society. (**C**) Molecular separation via DESI TWIMS MS, reprinted with permission from Towers et al, Optimised Desorption Electrospray Ionisation Mass Spectrometry imaging (DESI-MSI) for the Analysis of Proteins/Peptides from Tissue Sections on a Travelling Wave Ion Mobility Q-ToF, Analytical Chemistry, Copyright 2018, American Chemical Society.

Top-down protein analysis from tissue sections using LESA was first reported in 2014 for a 14 kDa protein, a (putative) liver disease biomarker from non-alcoholic steatohepatitis (NASH) tissue sections [41]. Subsequently, tissue imaging was reported from mouse brain (15 proteins) and liver (24 proteins), with FAIMS separation leading to significant improvements (34 and 40 proteins, respectively) [42]. Greater improvements were later reported for kidney tissue, which is otherwise dominated by haemoglobin signals [43], using the same (planar geometry) FAIMS device. Ion mobility separation afforded a 6- or 16- fold increase in the number of intact proteins for a single FAIMS condition, or a multi-step (multiple conditions) FAIMS experiment, in comparison with no ion mobility. Cyclic ion mobility provides improved coverage on a similar scale [44]. The use of a clindrical geometry FAIMS device, combined with the multi-step approach, showed improvements between 10- and 20-fold for brain, or testes and kidney tissue, compared with no FAIMS [45], as shown in Figure 3A, with much greater proteome coverage; 975, 981, and 249 for testes, kidney, and brain tissue, respectively. This improvement demonstrates a significant step for *in situ* omics, on a comparable scale to traditional chromatographic approaches. FAIMS additionally improves signal-to-noise ratios; recently this benefit was described for the first time for native LESA of protein assemblies up to 150 kD from standards and kidney tissue protein RidA (43 kDa) [46], see Figure 3B.

Native LESA, where folded proteins and non-covalent complexes are extracted using ammonium acetate buffered solutions, has also been demonstrated. In 2015, heamoglobin tetramers were extracted from dried blood spot cards [47] and tissue [48], where contact-LESA proved particularly useful. Data were acquired on Synapt instrumentation, exploiting the extended mass range (verses Orbitraps). The approach enables extraction and analysis of complexes up to 800 kDa from purified proteins and ligand binding can be probed [49], see Figure 5A, with careful control of the backing pressure proving particularly beneficial to transfer these ions. Refolding of proteins has also been demonstrated from purified standards spotted in denaturing solvents and sampled using ammonium acetate [50]. Yan et al. further showed that folded proteins, in low charge states, can be extracted using typically denaturing extraction solvents (acidified methanol) if the sample is kept at cryogenic conditions (Cryo-LESA) [51], see Figure 5B; five proteins were studied from tissue with charge states and ion mobility drift times comparable to extraction in ammonium based solvents at ambient temperature.

Imaging of folded intact proteins was reported, at 1 mm resolution, in 2019. Eleven proteins were imaged across murine brain between 5 and 14 kDa, and a 14 kDa protein alongside the 64 kDa haemoglobin tetramer in liver tissue [52]. This time, travelling-wave ion mobility mass spectrometry (TWIMS, available on Synapt instrumentation) was incorporated, with *in situ* collision cross-section (CCS) measurements made (by comparison CCS calibrant measurements from purified standards) of six proteins 5 and 15 kDa across the tissue sections, with measurements in agreement with published reports and purified standards [52]. Although this is a low-resolution imaging approach, the methods were ground-breaking for native imaging, demonstrating that the proteins were folded in a native state. High-resolution analysis of proteins up to 70 kDa using an extended mass range Orbitrap was reported in the same year [53], see Figure 5C. *In situ* ligand binding (non-covalently bound protein–drug complexes) has also been demonstrated on Orbitrap instrumentation from drug dosed rats (Figure 6A), benefitting from selected ion monitoring [54]. Despite the huge strides made for the analysis of native proteins and non-covalent complexes from tissue samples, until recently very little has been reported from bacterial colonies. However, electroporation followed by LESA sampling with ammonium buffered solvent systems recently enabled detection of superoxide dismutase homodimers up to 50 kDa from *E. coli* [55].

### Desorption electrospray ionisation (DESI)

Ionisation is achieved by electrospraying a charged solvent solution at a surface, with desorbed ions captured via an extended capillary [56], see Figure 1C. The detection of (denatured) protein standards up to 66 kDa was first reported in 2007, however molecular weight calculations were only reliable for proteins up to 17 kDa, with sensitivity limits ranging from 4 to 100 ng [57]. The use of solvent additives such as ammonium bicarbonate or serine can improve signal-to-noise ratios [58,59].

**Figure 4:**
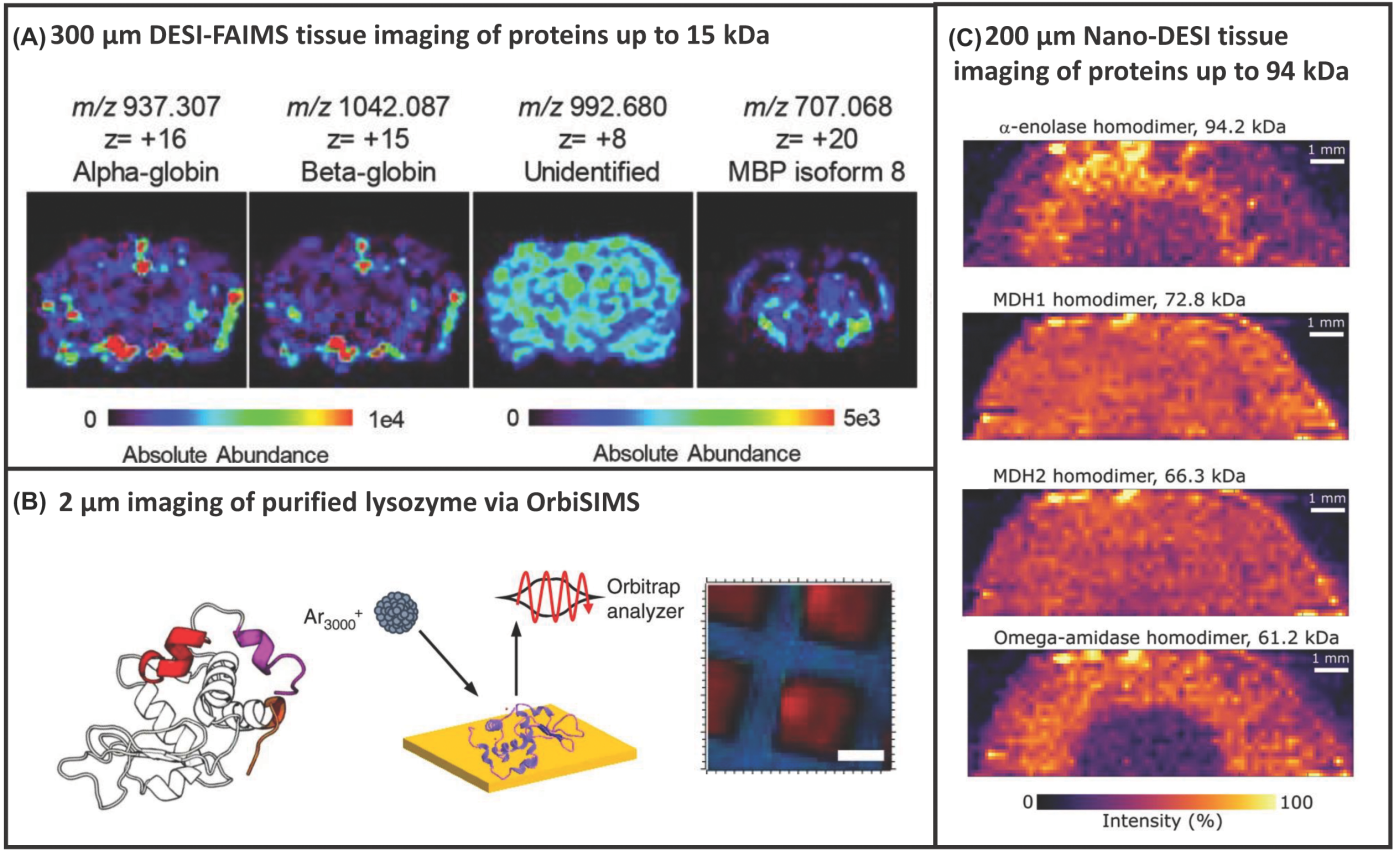
Current state of the art in protein imaging (**A**) 300 µm tissue imaging via DESI FAIMS MSI, reprinted with permission from Garza et al., Desorption Electrospray Ionisation Mass Spectrometry Imaging of Proteins Directly from Biological Tissue Sections, Analytical Chemistry, Copyright 2018, American Chemical Society. (**B**) About 2 µm protein imaging via OrbiSIMS, reprinted with permission from Kotowska et al, Protein identification by 3D OrbiSIMS to facilitate in situ imaging and depth profiling, Nature Communications, 2020, open access through a Creative Commons Licence. (**C**) About 200 µm tissue imaging via nano-DESI FAIMS MSI, reprinted with permission from Hale et al., Native Ambient Mass Spectrometry Enables Analysis of Intact Endogenous Protein Assemblies up to 145 kDa Directly from Tissue, Analytical Chemistry, Copyright 2022, American Chemical Society.

However, direct analysis from biological surfaces was not described until more recently. Analysis of intact proteins from complex samples such as tissue is not straightforward, requiring ion mobility separation to improve sensitivity of these ions in the presence of other (often more) abundant species such as lipids among other modifications. Garza et al. reported intact protein DESI imaging from tissues at 300 μm spatial resolution, with FAIMS described as a key aspect of their workflow, see Figure 4A. This enabled detection of 16 proteins, up to approximately 15 kDa [60]. Some proteins were detected with sufficient sensitivity to perform top-down MS/MS with sequence coverages ranging from 16 to 52%. Towers et al. also reported DESI imaging facilitated by ion mobility separation (TWIMS), see Figure 3C, in 2018 [61], at 150 μm spatial resolution. Tissue washing with ethanol or chloroform (to remove abundant lipids), modifying the voltage application to the emitter only (rather than the spray body) and using a heated capillary inlet (up to 430ᵒC) were beneficial for protein analysis. A total of seven proteins 5–16 kDa were detected.

**Figure 5:**
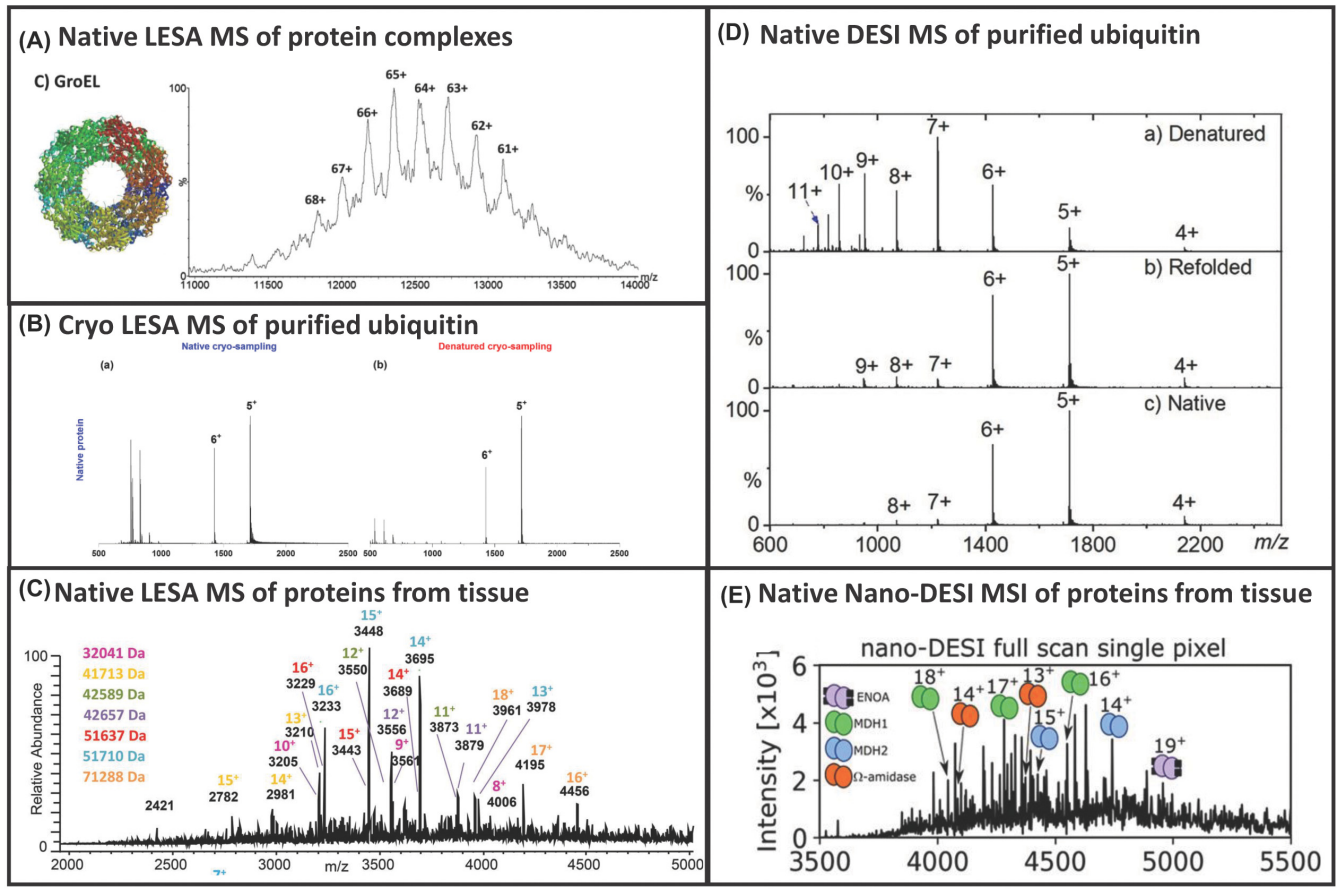
Native MS of intact proteins and complexes (**A**) Native LESA of protein complexes up to 800 kDa reprinted with permission from Mikhailov et al., Liquid extraction surface analysis for native mass spectrometry: Protein complexes and ligand binding, Journal of the American Society for Mass Spectrometry, 2016, open access through a Creative Commons Licence. (**B**) Cryo-LESA of ubiquitin reprinted with permission from Yan et al., Probing Folded Proteins and Intact proteins by Desorption Electrospray Ionisation Mass Spectrometry, Analytical Chemistry, Copyright 2021, American Chemical Society. (**C**) Native LESA of tissue proteins up to 70 kDa reprinted with permission from Griffiths et al, Direct mass spectrometry analysis of protein complexes and intact proteins up to >70 kDa from tissue, Analytical Chemistry, 2019, Copyright 2019, American Chemical Society. (**D**) Native DESI of purified ubiquitin reprinted with permission from Yan et al., Probing Folded Proteins and Intact Protein Complexes by Desorption Electrospray Ionisation Mass Spectrometry, Journal of the American Society for Mass Spectrometry, 2021, open access through a Creative Commons Licence. (**E**) Native Nano-DESI of protein complexes from tissue reprinted with permission from Hale et al, Native Ambient Mass Spectrometry Enables Analysis of Intact Endogenous Protein Assemblies up to 145 kDa Directly from Tissue, Analytical Chemistry, Copyright 2022, American Chemical Society.

**Figure 6:**
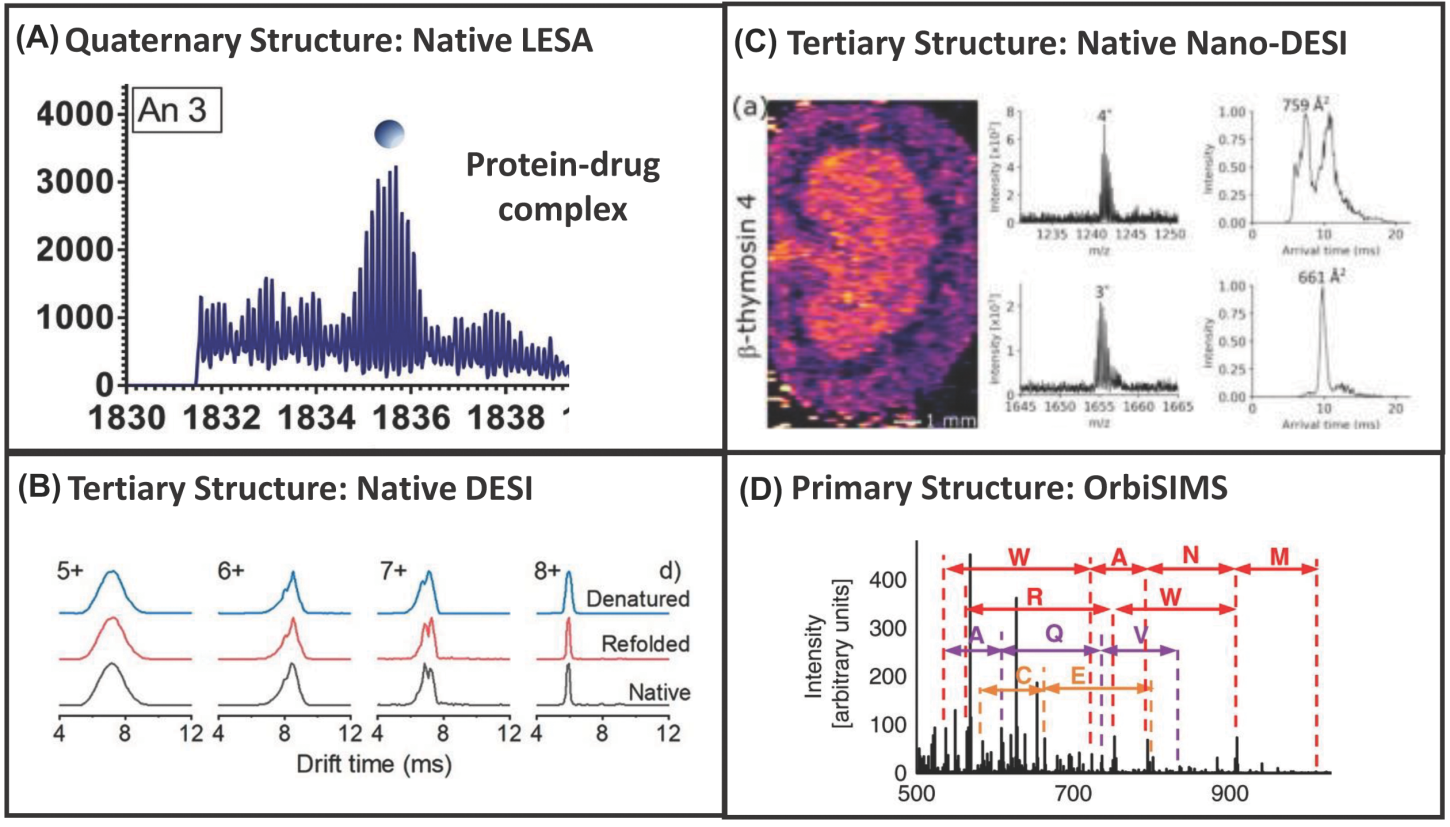
Probing protein structure (**A**) Native LESA MS of protein-drug binding reprinted with permission from Illes-Toth et al., Mass Spectrometry Detection and Imaging of a Non-Covalent Protein–Drug Complex in Tissue from Orally Dosed Rats, Angewandte Chemie International Edition, 2022, open access through a Creative Commons Licence. (**B**) Native DESI TWIMS of folded proteins reprinted with permission from Yan et al., Probing Folded Proteins and Intact Protein Complexes by Desorption Electrospray Ionisation Mass Spectrometry, Journal of the American Society for Mass Spectrometry, 2021, open access through a Creative Commons Licence. (**C**) Native Nano-DESI TWIMS of folded tissue proteins reprinted with permission from Hale et al, Simultaneous spatial, conformational, and mass analysis of intact proteins and protein assemblies by nano-DESI travelling wave ion mobility mass spectrometry imaging, international Journal of Mass Spectrometry, 2021, Rights Link. (**D**) Primary protein structure fragments via GCIB OrbiSIMS, reprinted with permission from Kotowska et al, Protein identification by 3D OrbiSIMS to facilitate in situ imaging and depth profiling, Nature Communications, 2020, open access through a Creative Commons Licence.

Native DESI of purified standards of proteins ranging from 8 to 800 kDa spotted on to glass slides has also been reported using ammonium acetate buffered sampling solvents. Ambrose et al. demonstrated it is possible to ionise non-covalent complexes, lipid–protein binding, and drug–protein binding [62]. The inclusion of detergent in the spray solvent was beneficial for native analysis of membrane proteins and minimising the transfer capillary length was recommended. Separately, charge state distributions indicative of folded protein structures, and refolding of denatured proteins were indicated by Yan et al. [63], see Figure 5D. Modifications that facilitated analysis included a heated capillary inlet, first described by Towers et al. However, top-down MS/MS, the measurement of CCSs, and imaging, has not been described via native DESI to date, despite charge state distributions being monitored, see Figure 6B. These studies clearly demonstrate promise of the approach for intact protein imaging from tissue. However, the number of modifications required to set up the DESI for protein analysis will perhaps be off-putting. Furthermore, DESI is commercially available for coupling to just one specific vendors’ instrumentation and so its implementation is limited unless you are willing to build your own. This comes with its own challenges that will understandably limit uptake of the approach.

### Flowprobe

Analytes are desorbed from a surface via continuous liquid flow sampling, which is then aspirated away from the surface via a capillary within the probe (see Figure 1D) before electrospraying into the mass spectrometer [64]. Imaging can be achieved either by discrete spot sampling or rastering across a sample surface. Imaging of proteins 4–12 kDa at 630 μm spatial resolution was reported by Fieder et al. in 2016 using spot-mode analysis using acetonitrile-based solutions [65]. Four proteins were identified from tissue via top-down MS/MS with sequence coverage ranging from 21 to 33%. Raster-mode imaging, which offers speed/throughput benefits, at 600 µm spatial resolution, was reported in 2017 with three proteins detected using methanol-based solvents [66]. Although these studies show some promise for the Flowprobe approach, subsequent imaging studies published via DESI and nano-DESI supersede its capability regarding spatial resolution (600 vs. 200 μm, respectively). It is, therefore, unsurprising that this has not seen uptake in the field.

### Nano-desorption electrospray ionisation (Nano-DESI)

Analytes are desorbed from a surface via continuous liquid flow sampling, aspirated away from the surface via a capillary before they are electrosprayed into the mass spectrometer, similar to Flowprobe MS. However, the geometry is different, the liquid junction is between two capillaries, called the solvent bridge, and the analysis surface, see Figure 1E. This technique was first demonstrated by Roach et al., showing detection of a 12 kDa protein standard [67]. Nano-DESI also allows direct detection and MS imaging of proteins in tissue sections; in 2015 Hsu et al. reported protein imaging up to 15 kDa from mouse brain at ∼200 μm spatial resolution [68]. Recently, multiplexed proteoform imaging MS (PiMS) has been described at sub-80 µm spatial resolution for annotation of up to 400 proteoforms up to 70 kDa from tissue sections [69]. Furthermore, the spatial resolution has been improved to 5 μm for intact proteins up to 14 kDa, with pneumatic-assisted transmission geometry to improve limits of detection and FAIMS to increase protein coverage (from 11 to 33) [70]. Furthermore, improvements in the microfluidic probe have been suggested for improved throughput [71]. A number of different myelin basic protein proteoforms (different post-translational modifications) have been reported using this approach for imaging of brain tissue [72].

Recently, several advances have been made in this field for the analysis of native (folded) proteins and non-covalent complexes. They demonstrated nano-DESI analysis of native intact proteins up to 145 kDa and protein assemblies up to 42 kDa directly from tissue and imaging of proteins up to 94 kDa at 200 μm spatial resolution on Orbitrap-based instrumentation [73,74], see Figure 4C. Eight protein assemblies were identified in kidney tissue [74], representing significant spatial resolution improvements over native-LESA and a significant increase in the highest mass ions detected by nano-DESI in comparison with previous reports. Ligand bound protein complexes and metal bound complexes (rather than protein–protein complexes) from brain tissues have also been reported using the nano-DESI approach [75]. Detection of a 113 kDa membrane protein has also been demonstrated from lens tissue without any sample pre-treatment, representing a significant advancement, and benefitting from the inclusion of an MS-compatible detergent [76]. Finally, native nano-DESI imaging of ten proteins ranging from 5 to 42 kDa has been reported (Figure 5E), alongside simultaneous CCS measurements [77], see Figure 6C, improving on the spatial resolution previously described for native LESA. These studies clearly demonstrate promise of the nano-DESI approach for native protein imaging from tissue. However, it is not a commercially available ion source, which will understandably limit uptake of the approach.

### Orbitrap secondary ion mass spectrometry (OrbiSIMS)

Ionisation is achieved by irradiating a surface/sample with a primary ion beam, leading to the expulsion of secondary ions from the surface, which are then analysed, see Figure 1F. SIMS has historically been used for elemental analysis, yet the introduction of gas cluster ion beam (GCIB) sources [78] and new instrument configurations have improved capability for biological analytes. GCIBs offer softer ionisation and therefore less fragmentation, allowing analysis of larger biomolecules. Proteins up to 12 kDa have been detected using GCIBs on time-of-flight SIMS instrumentation [79], and characterised from digests [80]. However, top-down MS/MS, imaging nor depth profiling were described until recently. The OrbiSIMS is a new tool for surface and depth analysis, combining high-spatial resolution of SIMS (∼2 μm) with the high mass-resolving power of an Orbitrap analyser and includes an argon (Ar_3000_^+^) GCIB [81]. Kotowska et al. analysed 16 different protein standards, ranging in size from 6 to 272 kDa, and described *in situ* analysis of intact proteins up to 50 kDA from human skin using the OrbiSIMS with the GCIB [82]. Although proteins were not digested in this study, it should be noted that the characteristic ions are not intact masses, but characteristic fragment ions. Proteins were further characterised via top-down MS/MS with sequence coverages ranging from 5 to 53%, see Figure 6D. Furthermore, intact proteins were imaged at a lateral resolution of 10 μm, see Figure 4B, a huge improvement over the capability of other approaches. Lysozyme and insulin could also be identified within a mixture via the OrbiSIMS approach, allowing for the identification of both proteins simultaneously. However, only three proteins were detected in the (more complex) tissue sample, demonstrating the current limitation of the approach. Furthermore, the analysis of native (folded) proteins has not been demonstrated via this approach and would probably prove challenging owing to the high energy nature of the ionisation. In addition, SIMS offers the opportunity to probe chemical localisation in 3D by depth profiling, e.g., through different skin layers. Kotowska et al. also show distributions of different skin proteins (using characteristic protein sequence markers) in the dermis, epidermis and stratum corneum. Whilst only relatively small fields of view can be imaged at a time (500 μm^2^), this approach clearly has real promise for the future of protein surface analysis and imaging and adds another dimension to spatial analysis.

## Perspective

Intact protein analysis via surface sampling approaches is experiencing some very exciting developments. Although MALDI has historically been coupled to time-of-flight instrumentation, to access high *m/z* for singly charged proteins, fundamental method developments demonstrated in liquid AP-MALDI (namely the generation of multiply charged ions that can be detected at lower *m/z*) provide exciting opportunities for analysis using high-resolution trapping instrumentation, and for improved top-down identification using this approach. Similarly, native protein analysis has typically been performed on time-of-flight instrumentation, to access lower charge states of folded proteins at high *m/z*. The introduction of extended mass range Orbitrap instruments offer exciting opportunities for intact protein and non-covalent complex analysis alike, as demonstrated by some of the literature reviewed herein. Regarding instrumentation, the inclusion of ion mobility separation in your workflow should be given serious consideration for *in situ* proteomics, benefiting improved sensitivity, improved signal-to-noise ratios and/or molecular separation, reducing interference from abundant background or other analyte ions.

With respect to imaging, Nano-DESI offers the current state-of-the art at 200 μm spatial resolution and is likely to remain for macroscale imaging of whole tissue sections. New instrumentation such as the OrbiSIMS offers exciting new avenues for in situ protein imaging at high resolution (2 μm), for microscale regions of interest. Remarkably, OrbiSIMS also offers structurally characteristic fragment ions afforded by the ionisation process. Whilst spectra will be complex and future efforts need to focus on developing databases for interpretation, the potential should not be under-estimated. SIMS also offers 3D depth profiling information, which is a new avenue in the intact protein area. Whilst liquid AP-MALDI may one day offer ambient imaging at high spatial resolution (10–20 μm), it seems the methods have not yet overcome practical hurdles for imaging applications. Native protein analysis remains an exciting burgeoning field; while LESA offers sensitivity for probing protein primary (via top-down MS/MS), secondary (combination of top-down MS/MS and CCS measurements), tertiary (through CCS measurement), and quaternary (ligand binding) structure, Nano-DESI offers high spatial resolution imaging, complementing one another.

Mass spectrometry imaging and native *in situ* proteomics remain key topics for future study. Liquid AP-MALDI and OrbiSIMS represent exciting new approaches for intact protein surface analysis. It is anticipated that investigating the benefits of additional ion mobility modalities will also prove valuable in the future.

## Summary

- Recent developments in ionisation offer further options for surface analysis and imaging using high resolution mass spectrometry.
- Ion mobility separation provides numerous advantages, namely molecular separation, improved signal-to-noise ratios, and the opportunity to probe protein conformation (tertiary structure).
- Native surface MS approaches provide a route to probing quaternary protein structure *in situ.*

## Open Access

Open access for this article was enabled by the participation of University of Nottingham in an all-inclusive *Read & Publish* agreement with Portland Press and the Biochemical Society under a transformative agreement with JISC.

## Abbreviations

CCS: collision cross-section
DESI: desorption electrospray ionisation
ESI: electrospray ionisation
GCIB: gas cluster ion beam
ISD: in-source decay
LESA: liquid extraction surface analysis
MALDI: matrix-assisted laser desorption/ionisation
TFA: trifluoroacetic acid
